# Significance of CCL2, CCL5 and CCR2 polymorphisms for adverse prognosis of Japanese encephalitis from an endemic population of India

**DOI:** 10.1038/s41598-017-14091-8

**Published:** 2017-10-20

**Authors:** Purvita Chowdhury, Siraj Ahmed Khan

**Affiliations:** Arbovirology division, Regional Medical Research Centre, NE Region, ICMR, Dibrugarh, 786001 Assam India

## Abstract

Japanese encephalitis (JE) is a major contributor for viral encephalitis in Asia. Vaccination programme has limited success for largely populated JE endemic countries like India and disease exposure is unavoidable. Involvement of chemokines and its co-receptors for adverse prognosis of JE have been documented both *in vitro* and *in vivo*. Identification of the genetic predisposing factor for JE infection in humans is crucial but not yet established. Therefore, we investigated the association of single nucleotide polymorphisms (SNPs) in chemokines (*CCL2* and *CCL5*) and its co-receptors (*CCR2* and *CCR5*) with their protein level for JE. The study enrolled 87 symptomatic JE cases (mild: severe = 24:63) and 94 asymptomatic controls. Our study demonstrated that *CCL2* (rs1024611G), *CCL5* (rs2280788G) and *CCR2* (rs1799864A) significantly associated with JE (Odds ratio = 1.63, 2.95 and 2.62, respectively and P = 0.045, P = 0.05 and P = 0.0006, respectively). The study revealed that rs1024611G allele was associated with elevated level of CCL2. CCL5 elevation associated with JE mortality having a Cox proportional hazard of 1.004 (P = 0.033). In conclusion, SNPs of chemokine *viz*. *CCL2* (rs1024611G) and its receptor *CCR2* (rs1799864A) significantly associated with JE which may serve as possible genetic predisposing factor and CCL5 protein level may act as marker for disease survival.

## Introduction

Japanese encephalitis (JE), a mosquito borne disease, is one of the major viral encephalitis in Asia and parts of Western Pacific^1^. It is estimated that globally 67,900 people are infected with Japanese encephalitis virus (JEV) per year. Among the JE endemic countries, India is one of the largest contributors of the disease burden^2^. Persistent JE outbreaks since the last four decades have been reported from the northeast (NE) India, particularly the state of Assam which confers around 30–40% of the country’s total JE burden (http://nvbdcp.gov.in/Doc/je-aes-cd-May16.pdf).

Several mechanisms have been postulated for JE related morbidity and mortality but exact aetiology still remains unclear. Differential phenotypic spectrum upon JE infection seems a unique feature for the disease morbidity where it varies from asymptomatic to symptomatic^3^. Epidemiological survey documented that JE associated mortality ranges from 20–30% among the symptomatic individuals^4^. Studies have shown that host innate and adaptive immune response activated by JEV infection, are involved in controlling viral loads in infected humans^5^. Beside other flaviviruses *viz*. West Nile virus (WNV), Tick borne encephalitis virus (TBEV) and Dengue, the differential expressions of cytokines and chemokines are observed in mice brain and spleen during JEV infection^6–9^. Altered homeostasis of chemokines including *CCL2* (also known as MCP-1 [monocyte chemoattractant protein]), *CCL3* (also known as MIP-1α [macrophage inflammatory protein 1-α]), *CCL5* (also known as RANTES [regulated on activation, normal T cell expressed and secreted]), *CXCL8* (IL-8) and cytokines *viz*. tumour necrosis factor α (*TNFα*), interleukin-6 (*IL-6*), *IL-1β* have been associated with the disease outcome^10–14^. *In vitro* studies demonstrated expression of cytokines and chemokines in neural cells after JEV infection^11,15^. Although chemokine cascade has been identified as a most potent contributor in JEV infected mice, reports have not yet been substantiated in humans^6^.

Differential phenotypic outcome among the individuals upon infection may hypothesize the aetiology of the disease to be multi-factorial, complex and dependant on host immune as well as genomic architecture. Recent genetic studies among varied ethnic groups have identified multiple genetic markers for several viral and bacterial diseases^16,17^ with a series of functional potency. Studies are extremely limited for identifying the genetic predisposing factors that may quantify the risk for JE. Therefore, detection of genetic variants of chemokines and their functional impact on disease status is imperative for deeper understanding of the disease mechanism.

Hence, in this present study, we have designed a case control association study to map functionally important single nucleotide polymorphisms (SNPs) of the candidate genes *viz. CCL2*, *CCL5*, *CCR2* and *CCR5* with morbidity of JE. Further, this study aims to evaluate the chemokine level and its interaction with genomic polymorphism to understand the differential phenotypic outcome of the disease.

## Results

Vaccination status and gender were matched among the study subjects, though older individuals were predominantly affected by severity of the disease (P = 0.003) (Supplement Table S1). Incidence of fatal outcome was significantly higher among the severe JE cases compared to mild cases (P = 0.005).

### Association of the SNPs of chemokines and their co-receptors for JE

**Figure 1:**
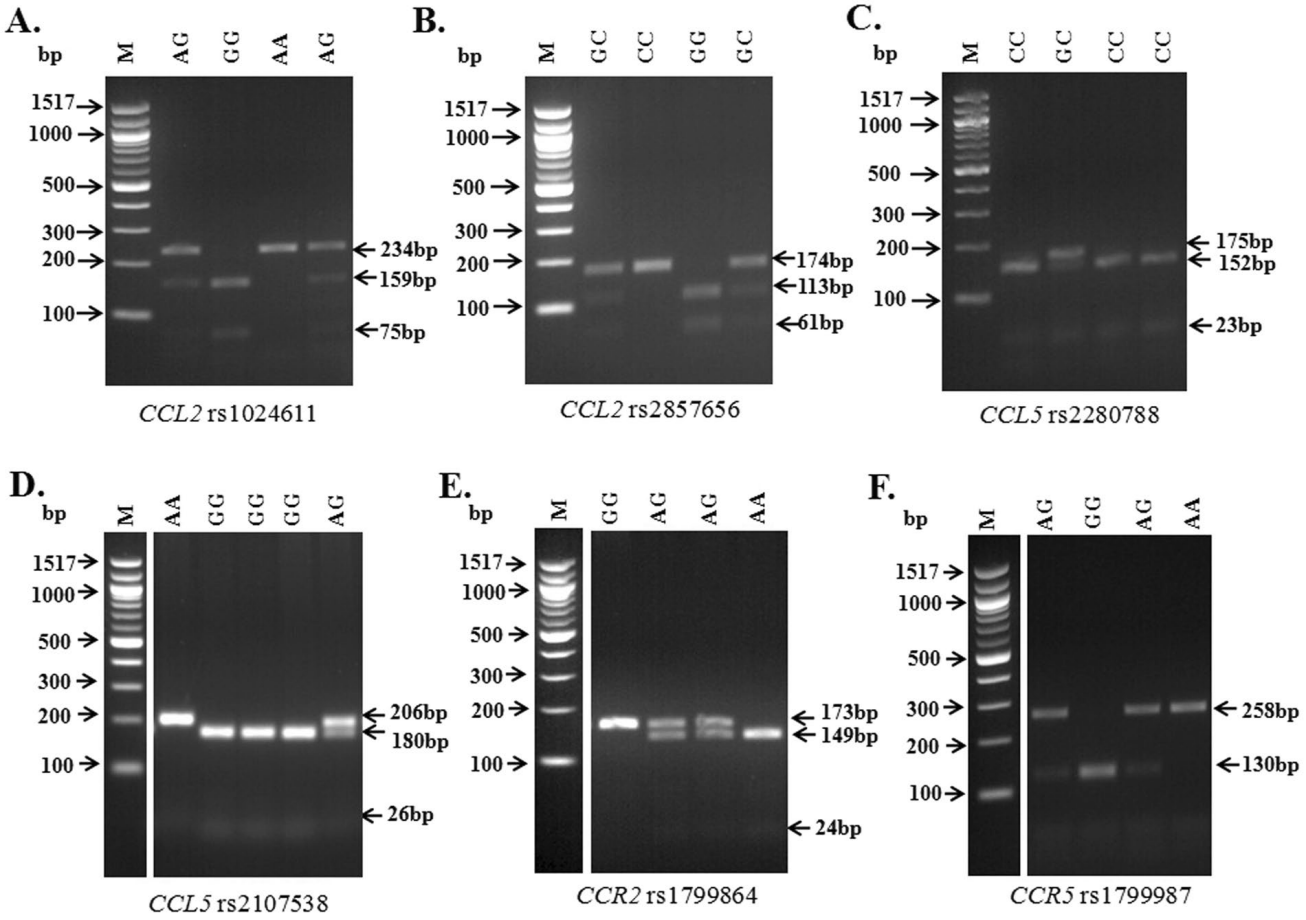
Genotyping of all the studied SNPs in agarose gel electrophoresis. (**A**) represents *CCL2* rs1024611A/G where AA homozygote was identified by a single 234 bp fragment, GG homozygote was characterized by 159 bp and 75 bp while AG heterozygote was identified by 234 bp, 159 bp and 75 bp fragments; (**B**) represents *CCL2* rs2857656G/C where CC genotype was identified by uncut 174 bp band, GG was identified by two fragments of 113 and 61 while CG heterozygote was characterized by 174 bp, 113 bp and 61 bp; (**C**) represents *CCL5* rs2280788C/G where CC genotype was identified by 152 bp and 23 bp fragments while for GC heterozygote, three bands at 175 bp, 152 bp and 23 bp was observed; (**D**) represents *CCL5* rs2107538G/A where an uncut single band of 206 bp corresponded to AA homozygote, 180 bp and 26 bp corresponded to GG homozygote while presence of three bands at 206 bp, 180 bp and 26 bp was identified as AG heterozygote; (**E**) denotes *CCR2* rs1799864A/G where an uncut single band of 173 bp was identified as GG, 149 bp and 24 bp was characterized by AA homozygote while AG heterozygote was identified by 173 bp, 149 bp and 24 bp fragments; (**F**) represents *CCR5* rs1799987A/G where AA genotype was characterized by 258 bp fragment, GG genotype was identified by 130 bp fragment while for AG heterozygote bands at 258 bp and 130 bp was observed. Images (**D**), (**E**) and (**F**) are vertically sliced images of juxtaposed lanes that were non-adjacent to the molecular marker and are delineated with white space. Gel images shown here are cropped images and full length gels are presented in Supplementary Fig. S7. Abbreviations: M, molecular size marker; bp, base pairs.

Genotyping was done for the entire group of 87 cases (JE patients) and 94 controls. Electrophoretic gel images for genotyping each studied SNPs are shown in Fig. 1. No significant departure from Hardy Weinberg equilibrium (HWE) was observed at rs1024611A/G (*CCL2*-2518), rs2857656G/C (*CCL2* -362), rs2280788C/G (*CCL5* -28), rs2107538A/G (*CCL5* -403), rs1799864G/A (*CCR2* V64I) and rs1799987G/A (*CCR5* 59029) loci among the controls (Table 1).

**Table 1:** Genotype and allele distribution among cases and controls.

Gene rs id	HWE for controls χ^2^ (P value)	Genotype/Allele	Case (n = 87)	Control (n = 94)	Odds ratio (95% CI)	P value
CCL2 rs1024611	1.13 (P = 0.287)	AA	47	57	0.762 (0.42–1.37)	0.452
		AG	22	30	0.722 (0.37–1.38)	0.411
		GG	18	7	3.24 (1.28–8.2)	0.016*
		A	116	144	0.611 (0.38–0.96)	0.046
		G	58	44	1.636 (1.03–2.59)	0.046*
CCL2 rs2857656	2.34 (P = 0.126)	GG	56	60	1.023 (0.55–1.88)	1
		GC	21	27	0.789 (0.40–1.53)	0.505
		CC	9	7	1.434(0.51–4.03)	0.603
		G	133	147	0.951 (0.57–1.56)	0.899
		C	39	41	1.051 (0.63–1.72)	0.899
CCL5 rs2280788	0.07 (P = 0.791)	CC	75	89	0.35 (0.118–1.041)	0.072
		GC	11	5	2.57 (0.857–7.744)	0.115
		GG	1	0	—	
		C	161	183	0.33 (0.118–0.969)	0.05
		G	13	5	2.95 (1.031–8.47)	0.05*
CCL5 rs2107538	3.10 (P = 0.078)	GG	39	46	0.84 (0.472–1.521)	0.655
		AG	36	34	1.24 (0.684–2.268)	0.541
		AA	11	14	0.82 (0.353–1.934)	0.673
		G	114	126	0.96 (0.623–1.499)	0.911
		A	58	62	1.03 (0.666–1.603)	0.911
CCR2 rs1799864	2.34 (P = 0.125)	GG	48	74	0.33 (0.173–0.637)	0.0008*
		AG	27	17	2.03 (1.017–4.081	0.05
		AA	9	3	3.5 (0.915–13.382)	0.072
		G	123	165	0.40 (0.231–0.702)	0.001*
		A	45	23	2.62 (1.508–4.567)	0.0006*
CCR5 rs1799987	3.60 (P = 0.057)	GG	31	35	0.93 (0.509–1.710)	0.877
		AG	34	35	1.08 (0.593–1.971)	0.878
		AA	22	20	1.25 (0.627–2.499)	0.598
		G	96	105	0.97 (0.642–1.473)	0.916
		A	78	83	1.02 (0.678–1.556)	0.916

P values were calculated by chi square test and * denotes statistical significance at P < 0.05. Abbreviations: rs ID, reference SNP cluster ID; HWE, Hardy Weinberg Equilibrium; CI, confidence interval; P value, level of significance.

For three tested SNPs, significant differences in allele frequencies were observed across JE cases and control groups. In JE cases, the frequency of rs1024611G allele (*CCL2*) was significantly higher than the control group (case: 36.78% vs. control: 23.4%; OR = 1.636, 95%CI = 1.03–2.59, P = 0.04; Table 1). In case of *CCL5* rs2280788, our analysis revealed an increased frequency of G allele among cases than controls (JE cases: 7.47% vs. control: 2.65%; OR = 2.95, 95%CI = 1.031–8.47, P = 0.05; Table 1). The data revealed that the *CCL2* rs1024611G (OR = 2.337, 95%CI = 1.2–4.54, P = 0.017) and *CCL5* rs2280788G (OR = 5.22, 95%CI = 1.523–17.947, P = 0.01) significantly associated with mild phenotype compared to controls (Supplementary Table S2).

Genotype of *CCR2* rs1799864 documented as protected allele where the frequency of rs1799864G significantly reduced among the cases with an odds ratio of 0.40 (95% CI: 0.231–0.702, P = 0.001). Study further revealed that the risk of the disease significantly increased by 2.62 fold for rs1799864A of *CCR2* (95%CI = 1.508–4.567, P = 0.0006). Moreover, *CCR2* rs1799864A allele frequency was significantly higher among mild and severe JE cases than controls (mild JE cases: 54.16% vs. control: 36.50%, P = 0.01; severe JE cases: 26.22% vs. controls: 12.23%, P = 0.002) (Supplementary Table S2).

Among the six tested SNPs, the study did not reveal significant association for rs2857656G/C of *CCL2*, rs2107538G/A of *CCL5* and rs1799987G/A of *CCR5* genotypes. Moreover, genotype and allele frequencies of all the studied SNPs did not differ significantly among mild and severe JE cases.

### Combined allelic association between JE cases and controls

To evaluate synergic effect for alleles of associated SNPs, χ^2^ test was used. The study revealed that the individuals lacking the variant allele of *CCL2* rs1024611, *CCL5* rs2280788 and *CCR2* rs1799864, in combination, decreased the risk of the disease by 0.47 fold (95%CI = 0.253–0.878; P = 0.02) (Table 2). However, no significant difference of combined SNPs was observed across mild and severe JE cases.

**Table 2:** Frequency distribution of combined alleles among cases and controls.

Combination allele	Case n = 87 (%)	Control n = 94 (%)	OR (95% CI)	P value
All 3 SNPs are dominant	4 (4.59)	0	—	—
Two SNPs have dominant allele	19 (21.83)	11 (11.70)	2.108 (0.939–4.733)	0.074
One SNP has dominant allele	38 (43.67)	42 (44.68)	0.960 (0.533–1.727)	1
None of the SNP has dominant allele	24 (27.58)	42 (44.68)	0.471 (0.253–0.878)	0.020*

P values were calculated by chi square test and * denotes statistical significance at P < 0.05. Abbreviations: CI, confidence interval; P value, level of significance.

### Serum protein levels and its relation with genotype

**Figure 2:**
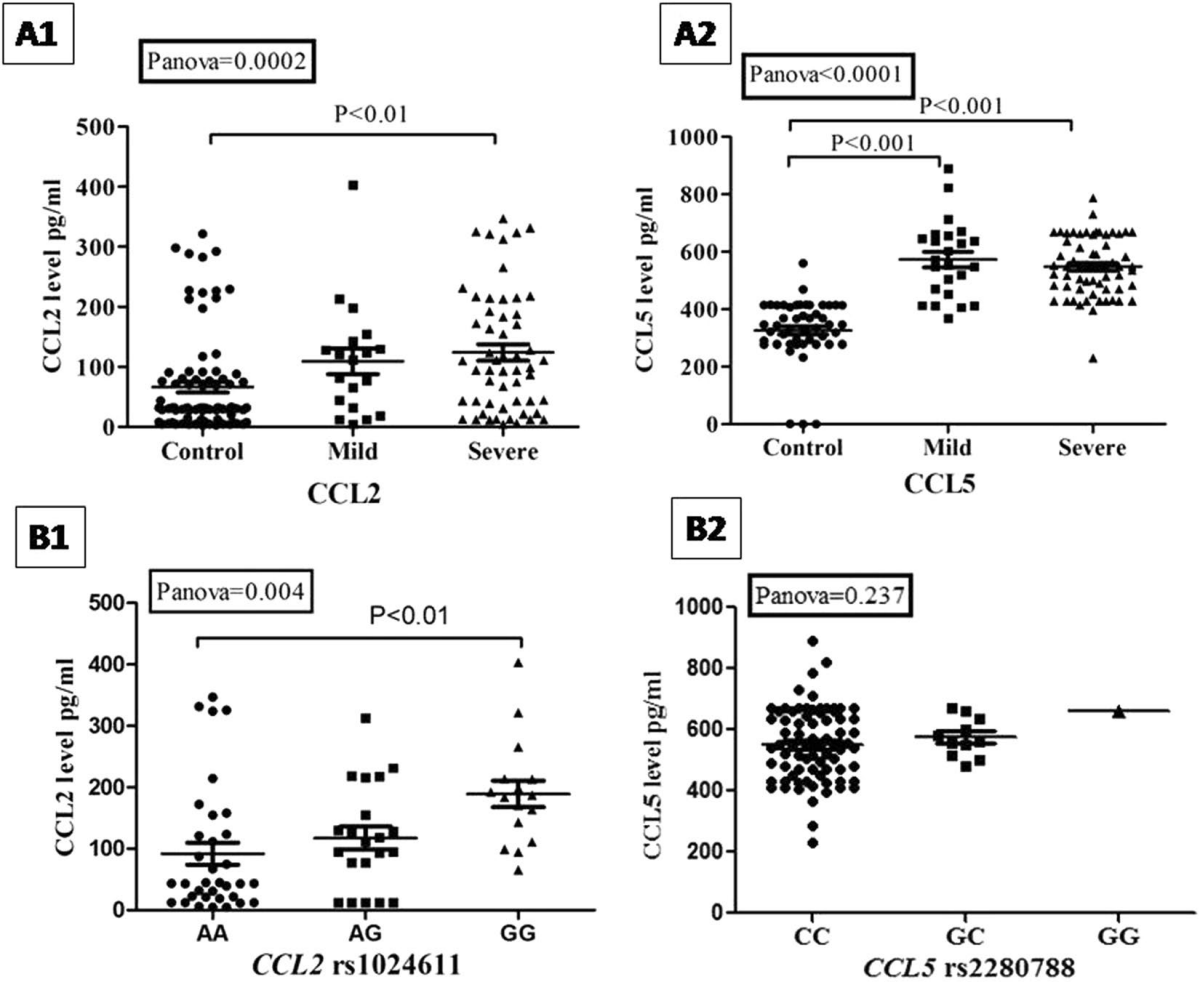
Summary of serum chemokine protein concentration among study groups (**A1** and **A2**) and according to genotypes of *CCL2* rs1024611 as well as *CCL5* rs2280788 (**B1** and **B2**) in cases. P values were calculated by one way ANOVA with Tukey’s multiple comparison test.

Serum concentration of CCL2 (sCCL2) and CCL5 (sCCL5) was significantly elevated among JE cases compared to controls (P = 0.0002 and P < 0.0001, respectively) (Fig. 2). Tukey’s test demonstrated that the mean concentration of sCCL2 was significantly elevated among severe JE cases than controls (P < 0.01) where the difference is insignificant between mild and severe phenotype of JE. In addition, the concentration of sCCL5 was significantly elevated among the cases compared to control group (P < 0.0001). We also performed non-parametric test for the data which showed similar trend of result (Supplementary Table S3). Figure 2 shows the P- values for parametric tests only.

The study further examined the differential distribution pattern of serum chemokine *viz*.CCL2 and CCL5 with respect to their genotypes among the JE cases. The study found concomitant elevation of sCCL2 concentration level with the increase of rs1024611G allele in a significant manner (P < 0.01) (Fig. 2). Additionally, Tukey’s test revealed that levels of sCCL2 was significantly higher (P = 0.004) among the cases with rs1024611GG genotype (189.10 ± 21.6 pg/ml) and lower among the rs1024611AA genotype (91.96 ± 16.29 pg/ml). However, we did not find any significant distributional difference of sCCL5 in context of rs2280788 genotype (550.8 ± 120.2 pg/ml in CC, 574.4 ± 70.3 pg/ml in GC and 661.6 pg/ml in GG; P = 0.237).

### Independent effect of SNPs and their interaction with JE

Binary logistic regression analysis of case- control data revealed that rs1799864A allele of *CCR2* significantly increased the disease risk with a log odds of 2.28 (P = 0.022) (Table 3). Concentration of sCCL2 and sCCL5 increased significantly the disease risk by 2.752 (P = 0.005) and 2.748 (P = 0.005) fold, respectively. Gene and protein interaction i.e. effect of corresponding variant allele and its subsequent elevation of sCCL2 levels significantly increased the disease risk with a log odds of 2.024 (P = 0.043). However, interactions among the other SNPs did not reveal any significant effect on the disease status.

**Table 3:** Association of chemokine proteins, SNP genotypes and its interaction to explain the disease status.

	Independent variable	Standard error	Z value	P value
Independent effect of serum protein level on disease status	CCL2	0.01883	2.752	0.00593*
	CCL5	0.03439	2.748	0.00599*
Independent effect of genotype on disease status	rs1024611	0.29991	1.053	0.292431
	rs2857656	0.27033	0.101	0.919405
	rs2107538	0.29961	1.442	0.149178
	rs2280788	1.30589	1.273	0.203187
	rs1799864	0.38339	2.284	0.022391*
	rs1799987	0.25315	−0.437	0.661915
Effect of genotype-genotype interaction on disease status	rs1024611: rs2280788	0.79428	0.444	0.65703
	rs1024611: rs1799864	0.41118	0.145	0.88486
	rs2280788: rs1799864	1.03252	−1.360	0.17398
Effect of genotype-protein interaction on disease status	rs1024611:CCL2	0.00341	2.024	0.0430*
	rs1799864:CCL2	0.00389	0.300	0.76408
	rs2280788:CCL5	1.151e + 01	0.004	0.99696

*Denotes P value significant at <0.05 level. Logistic regression was performed on case (n = 87) and control (n = 94) as dependant variable while the selected SNPs and protein were taken as the independent variable. It was used to fit a regression model to examine the effect of a protein level and genotype on the disease status. Abbreviations: Z value, regression coefficients divided by standard error; P value, level of significance.

### Survival analysis

The cases were followed up for one year post diagnosis. The overall survival rate at one year was 64.36% and the mean age of the fatal cases were 43 years (SD ± 19). The Kaplan Meier survival analysis did not reveal any significant difference among the genotypes of the associated SNPs (see Supplementary Fig. S4). The proportional death hazard significantly increased (P = 0.033) by 0.4% with increase of CCL5 protein even after considering together with other variables (age, gender, CCL2 protein, rs1024611, rs2280788 and rs1799864) in Cox proportional hazard model (Table 4).

**Table 4:** Cox proportional hazard analysis with putative prognostic factors for survival.

Selected factor	Hazard ratio for death (95% CI)	P value
CCL5	1.004 (1.0003–1.008)	0.033*
CCL2	0.998 (0.994–1.003)	0.557
Age	0.990 (0.969–1.011)	0.358
Gender	1.155 (0.485–2.749)	0.744
SNP1 (rs1024611)	1.035 (0.446–2.405)	0.934
SNP4 (rs2280788)	0.710 (0.146–3.445)	0.671
SNP5 (rs1799864)	1.297 (0.574–2.934)	0.530

P values were calculated by chi square test and * denotes statistical significance at P < 0.05. Abbreviations: CI, confidence interval; P value, level of significance.

## Discussion

Intra-cellular chemokine cascade may play a pivotal role for anti viral host defence^18^. Murine model and *in-vitro* studies have potentially demonstrated the activation of chemokine on JEV infection^6,15^. Identification of genomic markers for JE endemic area seems to be crucial but studies are substantially limited. Present study reveals that the variant allele of chemokines *viz. CCL2* and *CCL5* along with *CCR2* receptor are significantly associated with the disease.

Previous study demonstrated that chemokines are crucially involved in trafficking of peripheral monocytes, lymphocytes and neutrophils into the site of infection^18^. Among the varied group of chemokines, CCL2 is recognized as the most effective regulator of monocyte infiltration but its impact on JEV infection and its severity is still unexplored. During the severity of JEV infection, the virus usually infiltrates blood brain barrier (BBB) to CNS leading to influx of leukocytes. The infected cells in response trigger the production of cytokine and chemokine cascade which ultimately causes neuroinflammation. It has been also postulated that the elevated level of inflammatory cytokines *viz*. IL-6 and TNFα and chemokines *viz*. CCL5 and IL-8 may crucially be associated with adverse prognosis of JE^12,14^. As observed in our study, rs1024611G and rs2280788G at the promoter of *CCL2* and *CCL5* respectively, increases risk of the disease 1.63 and 2.95 folds significantly (Table 1). The study also demonstrated that chemokine concentration of sCCL2 and sCCL5 level significantly increased among the cases compared to controls (P = 0.0002 and P < 0.0001, respectively) (Fig. 2). Previous study demonstrated that the level of serum CCL2 significantly elevated upon administration of live attenuated JE vaccine in humans^19^. Recently, it was documented that expression of CCL2 elevated 1387.79 fold in JEV infected mice brain as one of the topmost hits from microarray dataset^6^. Elevated plasma concentration of CCL5 has been documented on JEV infected cases^14^. Repeated intra and inter species studies documented the crucial involvement of chemokine and cytokines for the adverse prognosis of JE. Single nucleotide polymorphisms (SNPs) found in *CCL2* and *CCL5* gene (chromosome 17), in *CCR2* and *CCR5* gene (chromosome 3) have been related to infectious diseases including West Nile virus^20,21^, dengue^22,23^, HIV^24,25^, Hepatitis C^26^ and tuberculosis^27^. However, there are no available reports which relate chemokine and its co-receptor SNPs to JE. Till date, genetic studies associating host genotype and JE are limited only to the analysis of TNFα and TLR3 polymorphisms^28,29^. rs1024611G and rs2280788G are associated with communicable diseases like pulmonary tuberculosis^30,31^ and Hepatitis C^32,33^. Not only in infectious diseases, transition of adenine (A) to guanine (G) at *CCL2* (−2518) locus i.e. rs1024611G signify important risks for variety of non communicable diseases. G allele of the locus is associated with coronary artery disease risk (OR = 2.2, 95% CI 1.25–3.92, P < 0.005)^34^ as well as systemic lupus erythematosus (OR = 4.2, 95% CI 1.8–9.6, P < 0.0001)^35^. Multi factorial genetic model reveals that rs2280788G may act as a predicting marker for the adverse prognosis of diabetic nephropathy^36^.

Our study revealed that G allele of *CCL2* promoter i.e. rs1024611 was significantly associated with elevated serum levels of the CCL2 where rs1024611AA individuals showed significantly reduced level of the same (91.96 ± 16.29 pg/ml) compared to variant homozygous genotype i.e. rs1024611GG (P < 0.01). Transition from A to G which is associated with elevated sCCL2 concentration may postulate its functional potentiality on transcription regulation. Furthermore, ChipSeq data reveals that the transcription factor *viz*. Myb and NFκB binds to *CCL2* promoter locus (rs1024611) and may modulate the gene regulation. A functional study reported that rs1024611, located in the promoter region, has been implicated with increased CCL2 expression^37^. Persistent replication of studies including the present observation suggests that rs1024611G could possibly result in an up-regulation of CCL2 and might boost leukocyte trafficking into CNS. This could trigger massive inflammation and may be related to adverse prognosis of the disease. On the other hand, very few functional studies on rs1799864 are available and report its involvement on the stability of CCR2 by a missense mutation from valine to isoleucine at the 64^th^ position of the protein^38^. Studies have documented that *CCR2* genetic deficiency may result in partial or entire absence of leukocytes in inflamed tissue in WNV and JEV in mice model^39,40^. Repeated observations postulate that rs1024611G, with 8 regulatory motifs, may act as a genomic marker with its strong functional potentiality.

The logistic regression analysis further revealed that rs1799864A allele significantly increases the disease risk 2.284 fold independently and the gene- protein interaction of rs1024611G and CCL2 protein level significantly associated with the odds of 2.024 (P = 0.043) for JE (Table 3). Further multinomial regression revealed rs1799864A independently associated with the mild phenotype of JE (P = 0.028) and gene-protein interaction for rs1024611G and CCL2 significantly explain the mild phenotype (P = 0.015) (Supplementary Table S5).

Our study also observed that rs1799864GG of *CCR2* was significantly less among the cases compared to controls that may provide protection by reducing the risk of JE by 0.33 fold for the present JE endemic population. Present study also reveals that the absence of variant allele of rs1024611, rs2280788 and rs1799864 synergistically reduces the disease risk 0.47 fold, P = 0.02. However, a previous study documented rs1799864A allele to provide protective effect among the human Pappiloma virus infected Brazilian patients^41^. Contradictory events for rs1799864 may implicate the heterogeneity among the population architecture (Supplementary Table S6). The allelic and genotype distribution in our study population were evidently different from other non JE endemic African and American population (Supplementary Table S6). Although the complete genomic architecture is still enigmatic for the population, the genotype frequencies of the tested associated SNPs were similar to the South Asian population reported in the 1000 Genomes Project Phase III database except rs1799864G that significantly decreased the disease risk. Additionally, it must be noted that the study population has close geographical proximity with the other highly JE endemic Southeast Asian countries. Fatality is the most adverse outcome that associated with mild and severe phenotype. When JEV evades the host’s primary immune response, it enters into the CNS and damages the non renewable neuronal cells. This may further induce severe inflammation causing encephalitis and death. According to the WHO reports, the case fatality has been recorded as 30% which is similar to our study where case fatality in JE patients is 35.63% (Supplementary Table S1). The study observed that 32% of mild JE cases and 67.7% from severe cases had a fatal outcome which is significantly high in severe cases (P = 0.005) (Supplementary Table S1). The study demonstrated that serum CCL5 significantly associated with the mortality of JE infection with Cox proportional hazards (P = 0.03) (Table 4).

In the present study, adults were observed to be predominantly suffering from JE. This could be attributed to regular childhood immunization programmes for the past 10 years in the study population. This phenomenon of changing age specificities of JE has been previously documented in other JE endemic areas with child immunizations^4^. Although a definite explanation is dubious, male preponderance among severe JE cases is well reflected in this study as well as previous studies^42,43^.

 No significant difference was observed when SNPs were compared among mild and severe JE cases. This could be probably explained with further longitudinal studies on respective chemokine and its receptor gene expression. Though the present pilot study is limited with its sample size, but replication of findings in a large cohort is extremely important for validation. As accepted, gene expression is governed by many regulatory factors rather than exclusively by promoter. Thereby, it is possible that other genetic influences may have a role in the prognosis of the disease which was not evaluated in our study.

In conclusion, the present findings suggests that *CCL2* (rs1024611G), *CCL5* (rs2280788C) and *CCR2* (rs1799864A) is associated with JEV infection and may act as a predictive marker for the adverse prognosis of JE in the population from northeast India. Moreover, rs1024611G (risk allele) is associated with increased serum CCL2 and may play a critical role in its adverse prognosis. Notably, CCL5 protein levels can be crucial marker for survival of JE as it is significantly associated with the fatal outcome.

## Materials and Methods

### Study cohort and diagnosis

Present cross sectional study comprised of 181 subjects (Case: 87 and Controls: 94). Cases were recruited from Assam Medical College & Hospital and other hospitals in Dibrugarh, India. The diagnosis of acute JE infection was based on clinical symptoms and serological positivity in accordance with WHO criteria^44^. The likelihood of cross reactivity with other circulating flavivirus *viz*. West Nile (WN) virus was considered. As a result, JE infected individuals nonreactive to WN specific IgM antibodies were confirmed as case. Cross-reactivity with dengue was not tested as the clinical symptoms are distinctly different from JE.

Cases were further stratified as mild and severe JE on the basis of their clinical symptoms as diagnosed by clinicians. Mild JE (n = 24) was characterized as JE patients who had acute fever, irritability, headache and muscle pain while patients with paralysis and encephalitis (altered mental status varying from confusion to coma) were considered as severe JE (n = 63). Asymptomatic serologically confirmed JE positive individuals from JE endemic area were considered as controls. People with other infections within one month, chronic inflammatory and autoimmune diseases were excluded from the study. Each case was followed up for one year and health status was obtained through structured questionnaire. The study was approved by the Institutional Ethics Committee of Regional Medical Research Centre, Indian Council of Medical Research (ICMR), Assam, India. Informed written consent from each participant was obtained prior to the study. Additionally, the study was carried out in accordance with Declaration of Helsinki and all methods were performed according to the recommended guidelines and regulations.

### Sampling

Venous blood was collected from study participants in EDTA vials (for genomic DNA isolation) and sterile plain vials for serum. The samples were stored at −80 °C until further processing.

### *In silico* annotation for SNP selection in promoter and coding region

The study used the UCSC Genome Browser and ChipSeq data to predict the functional potentiality of single nucleotide polymorphisms (SNPs) that are present in *CCL2*, *CCL5*, *CCR2* and *CCR5* promoter and coding regions in 1000 Genome datasets (http://www.1000genomes.org/home). Predictions about the functional consequences of its variant region and impact were assessed using HaploregV3^45^.

### Genotyping

Genomic DNA was isolated by QIAamp Blood DNA kit (Qiagen, USA; Cat no.: 51104) from each study subject as per manufacturer’s instructions from venous blood. Genotyping for the candidate SNPs of chemokine and chemokine receptor genes *viz*. rs1024611A/G (-2518) and rs2857656G/C (-362) of *CCL2*, rs2107538G/A (-403) and rs2280788C/G (-28) of *CCL5*, rs1799864A/G (V64I/+190) of *CCR2* and rs1799987G/A (-59029) of *CCR5* was performed by PCR-restriction fragment length polymorphism (RFLP)^46–48^. Details of SNP gene location, primer sequences, cycling conditions, and restriction enzymes (RE) with the amplicon size are given in Table 5.

**Table 5:** Details of PCR primers and conditions for genotyping of study SNPs.

Gene	rsID (location)	Primer (5′-3′)	Annealing temperature (°C)	Amplicon size (bp)	Restriction Enzyme	Allele size (bp)
CCL2	rs1024611 (−2518A/G)	F-TCTCTCACGCCAGCACTGACC	56	238	*Pvu*II	G = 159,75 A = 234
		R-GAGTGTTCACATAGGCTTCTG				
	rs2857656 (−362G/C)	F-GAGCCTGACATGCTTTCATCTA	58	174	*Hpy*188I	G = 113,61 C = 174
		R-TTTCCATTCACTGCTGAGAC				
CCL5	rs2280788 (−28C/G)	F-ACTCCCCTTAGGGGATGCCCG	55	175	*Hinc*II	C = 152,23 G = 175
		R-GCGCAGAGGGCAGTAGCAAT				
	rs2107538 (−403G/A)	F-CACAAGAGGACTCATTCCAACTCA	50	206	*RsaI*	G = 180,26 A = 206
		R-GTTCCTGCTTATTCATTACAGATCGTA				
CCR2	rs1799864 (V64I)	F-TTGGTTTTGTGGGCAACATGATGG	56	173	*BsaBI*	A = 149,24 G = 173
		R-CATTGCATTCCCAAAGACCCACTC				
CCR5	rs1799987 (59029 G/A)	F-CCCGTGAGCCCATAGTTAAAACTC	65	258	*Bsp*1286I	G = 130 A = 258
		R-TCACAGGGCTTTTCAACAGTAAGG				

Abbreviations: rs ID, reference SNP cluster ID; bp, base pair.

All PCRs were done in 20 μl reaction volumes using 2X Master Mix (Promega, Wisconsin, USA; Cat no.: M7505). PCR was performed using Applied Biosystems Veriti 96 well Thermal Cycler (California, USA). The amplified products were digested with 1.5U of specific restriction enzyme (New England Biolabs, Massachusetts, USA) as per manufacturer’s instructions and subsequently separated by electrophoresis on 3% agarose gel stained with 1 mg/ml ethidium bromide.

### Serum CCL2 and CCL5 protein assay

Protein concentration of CCL2 and CCL5 were determined from serum using commercially available ELISA kits as per manufacturer’s instruction (RayBio^®^, USA; Cat no.: ELH-MCP1-001 and ELH-RANTES-1). The minimum detectable quantity of CCL2 and CCL5 as per the used kit is 2 pg/ml and 3 pg/ml respectively.

### Statistical analysis

Age, gender, vaccination status and fatality were compared between JE (mild and severe) cases and controls using chi square (χ^2^) and two-tailed Student’s t-test. Genotypic frequency among cases and controls were compared byχ^2^ test. Hardy Weinberg equilibrium was analysed byχ^2^ test with P > 0.05 considered as equilibrium. Odds ratio (OR) was calculated using Fisher’s exact test at 95% confidence interval (95% CI). Protein level was analyzed by one way ANOVA and Tukey’s multiple comparison tests among the study groups *viz*. mild, severe and control. Data were presented in mean ± standard deviation of mean (SD). Logistic regression model was used to fit a regression model to examine the effect of chemokine protein level and genotypes on the disease (cases vs. control). The dependant variable i.e. cases and control was coded as 1 and 0 respectively. In addition to the independent SNP effect, combined or synergic effect was also tested to examine if risk alleles of the three associated loci could magnify the genetic risk usingχ^2^ test. The survival analysis for surviving and fatal JE cases was estimated using Kaplan Meier method. The possible predictive significance of chemokine proteins and SNPs on JE survival was assessed using Cox proportional-hazards modelling. The p-value < 0.05 was considered as significant. All the statistical analysis was conducted in R package (version 3.2) and GraphPad Prism 5.

### Data availability

All data generated or analysed during this study are included in this published article (and its Supplementary Information files).

## Electronic supplementary material

supplementary data
