# Supplementary material for: Significance of CCL2, CCL5 and CCR2 polymorphisms for adverse prognosis of Japanese encephalitis from an endemic population of India
Source: Sci Rep. 2017 Oct 20;7:13716. doi: 10.1038/s41598-017-14091-8 (PMC5651904; doi:10.1038/s41598-017-14091-8)

Significance of CCL2, CCL5 and CCR2 polymorphisms for adverse prognosis of Japanese encephalitis from an endemic population of India

Purvita Chowdhury[1], and Siraj Ahmed Khan[1,*]

[1]Arbovirology division, Regional Medical Research Centre, NE Region, ICMR, Dibrugarh-786001, Assam, India

***sirajkhanicmr@gmail.com**

Corresponding author: Siraj Ahmed Khan

Supplementary information

Supplementary Table S1. Demographic characteristics among cases and controls. P value for age was calculated by two tailed t-test and chi square test was used for gender, fatality and vaccination. * denotes significance at <0.05. Values are expressed as mean ± standard deviation and absolute numbers (%). Abbreviations: P value, level of significance; SD, standard deviation.

	**Severe (n=63)**	**Mild (n=24)**	**Control (n=94)**	**P value**
Age (in years, mean ± SD)	44.84 ± 20.17	42.13 ± 19.8	35.28 ± 15.65	0.003*
**Gender**<br>Male (%)<br>Female (%)	 43 (68.25%)<br>20 (31.74%)	 11 (45.83%)<br>13 (54.16%)	 55 (58.51%)<br>39 (41.48%)	 0.143
**Fatality** (%)	21 (67.74%)	10 (32.25%)	-	0.005*
**Vaccination history**<br>Not vaccinated<br>Vaccinated	 55<br>8	 21<br>3	 94	 0.98

Supplementary Table S2. Genotypic and allelic distribution among JE mild and severe cases compared to controls. Odds ratio was calculated by chi square test and * denotes statistical significance at P<0.05. Abbreviations: rs ID, reference SNP cluster ID; CI, confidence interval; P value, level of significance

**Type of JE**	**Gene rs id**	**Genotype/ Allele**	**Case**	**Control (n=94)**	**Odds ratio (95% CI)**	**P value**
Mild JE	CCL2	AA	11	57	0.549 (0.22-1.35)	0.247

**(n=24)**	rs1024611	AG	6	30	0.711 (0.25-1.97)	0.623
		GG	7	7	5.117 (1.58-16.48)	0.008*
		A	28	144	0.427 (0.21-0.83)	0.017
		G	20	44	2.337 (1.2-4.54)	0.017*
	CCL2 rs2857656	GG	15	60	0.669 (0.27-1.65)	0.48
		GC	7	27	0.653 (0.22-1.92)	0.461
		CC	2	7	4.142 (0.24-13.79)	0.024
		G	37	147	0.508 (0.25-1.0)	0.06
		C	11	41	1.966 (0.99-3.90)	0.06
	CCL5 rs2280788	CC	19	89	0.21 (0.056-0.811)	0.028
		GC	4	5	3.56 (0.876-14.457)	0.081
		GG	1	0	-	
		C	42	183	0.19 (0.055-0.656)	0.01
		G	6	5	5.22 (1.523-17.947)	0.01*
	CCL5 rs2107538	GG	7	46	0.42 (0.163-1.131)	0.108
		AG	13	34	2.08 (0.842-5.163)	0.160
		AA	3	14	0.816 (0.214-3.106)	1
		G	27	126	0.69 (0.361-1.354)	0.303
		A	19	62	1.43 (0.738-2.769)	0.303
	CCR2 rs1799864	GG	12	74	0.02 (0.105-0.692)	0.008*
		AG	9	17	2.71 (1.02-7.232)	0.053
		AA	2	3	2.75 (0.434-17.517)	0.581
		G	33	165	0.35 (0.162-0.768)	0.01*
		A	13	23	2.82 (1.3-6.14)	0.01*
	CCR5 rs1799987	GG	11	35	1.42 (0.576-3.537)	0.486
		AG	9	35	1.01 (0.4-2.553)	1
		AA	4	20	0.74 (0.227-2.412)	0.779
		G	31	105	1.44 (0.746-2.782)	0.327
		A	17	83	0.693 (0.359-1.339)	0.327
Severe JE **(n=63)**	CCL2 rs1024611	AA	36	57	0.865 (0.45-1.65)	0.74
		AG	16	30	0.726 (0.35-1.48)	0.474
		GG	11	7	2.629 (0.95-7.2)	0.073
		A	88	144	0.707 (0.42-1.17)	0.192
		G	38	44	1.413 (0.84-2.35)	0.192
	CCL2 rs2857656	GG	43	60	1.282 (0.64-2.54)	0.49
		GC	16	27	0.863 (0.41-1.77)	0.718
		CC	3	7	0.632 (0.15-2.54)	0.74
		G	102	147	1.293 (0.72-2.30)	0.392
		C	22	41	0.773 (0.43-1.37)	0.392
	CCL5	CC	56	89	0.44 (0.136-1.485)	0.224

	rs2280788	GC	7	5	2.22 (0.673-7.353)	0.224
		GG	0	0	-	
		C	119	183	0.46 (0.144-1.497)	0.233
		G	7	5	2.15 (0.667-6.941)	0.233
	CCL5 rs2107538	GG	8	46	0.83 (0.326-2.115)	0.816
		AG	23	34	1.01 (0.522-1.969)	1
		AA	32	14	1.07 (0.568-2.039)	0.871
		G	39	126	0.91 (0.561-1.479)	0.713
		A	87	62	1.09 (0.675-1.782)	0.713
	CCR2 rs1799864	GG	36	74	3.79 (0.941-15.267)	0.09
		AG	18	17	1.81 (0.848-3.866)	0.17
		AA	7	3	0.36 (0.178-0.727)	0.004*
		G	90	165	0.39 (0.216-0.710)	0.002*
		A	32	23	2.55 (1.408-4.620)	0.002*
	CCR5 rs1799987	GG	20	35	0.78 (0.399-1.540)	0.5
		AG	25	35	1.10 (0.575-2.136)	0.867
		AA	18	20	1.48 (0.708-3.091)	0.343
		G	65	105	0.84 (0.535-1.324)	0.489
		A	61	83	1.18 (0.754-1.867)	0.489

Supplementary Table S3: **Parametric and non-parametric test for serum concentration of chemokines CCL2 and CCL5**. One way analysis of variance (ANOVA) and Kruskal Wallis test was performed for parametric and non-parametric analyses. The data revealed similar significant values (P<0.05) in both parametric and non-parametric tests. The manuscript Fig. 2 depicts P-values for parametric tests only.

	**Parametric test (P-value)**	**Non-parametric test (P-value)**
**Serum protein concentration**		
CCL2 protein level	0.0002	0.0001
CCL5 protein level	<0.0001	<0.0001
**Genotype wise protein concentration**		
CCL2 rs1024611	0.004	0.0015
CCL5 rs2280788	0.237	0.441

Supplementary Figure S4: Kaplan Meier curve for *CCL2* rs1024611, *CCL5* rs2280788 and *CCR2* rs1799864.

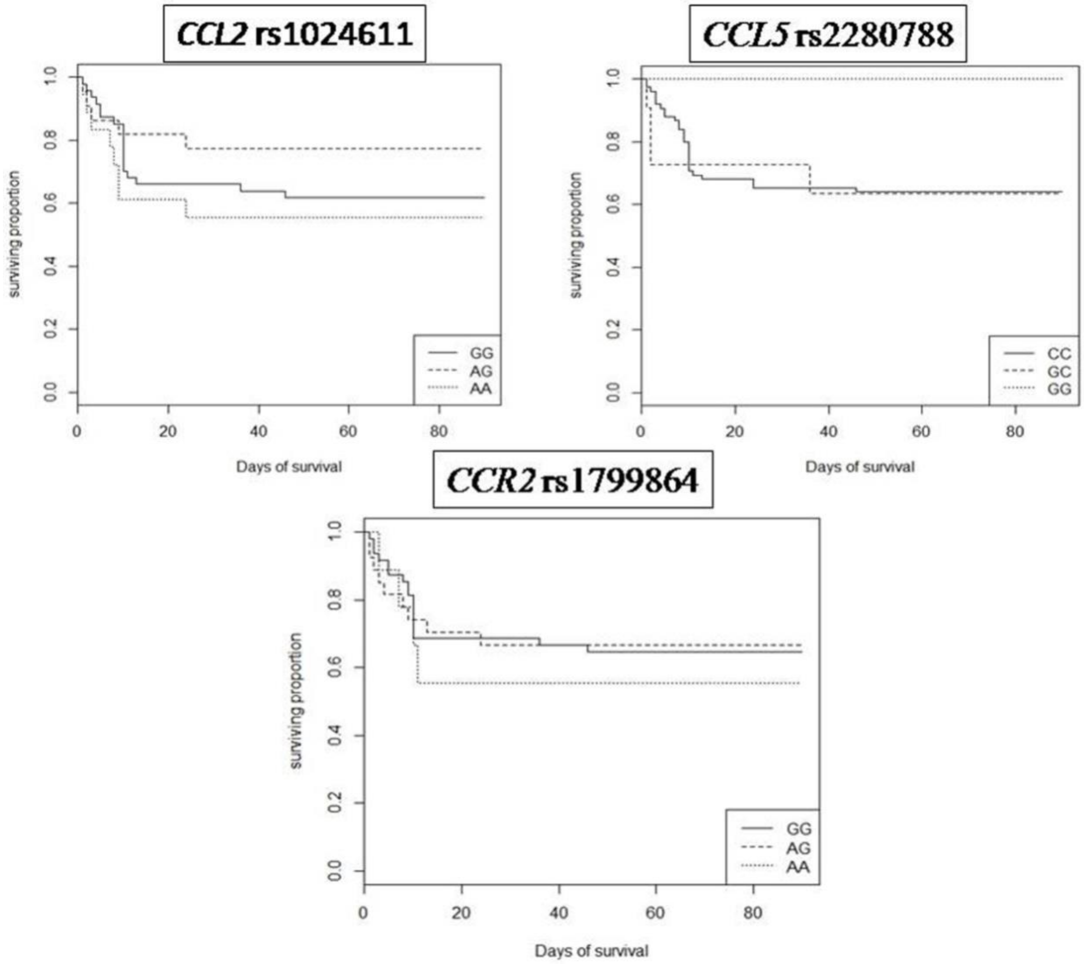

Supplementary Table S5: **Genetic factors and chemokine levels relating to clinical phenotypes of JE.** Multinomial Logistic Regression was used to examine the effect of chemokine protein level and corresponding genotype on the study groups (where 0 = control, 1=mild JE, 2 = Severe JE). This model explains the extent of effect the protein level and genotype independently or in interaction has on any of the disease group when compared with the baseline level (controls). SNP1=rs1024611; SNP4= rs2280788; SNP5= rs1799864; * indicate statistically significant at P value <0.05

Predictor	JE Status	Coefficients	P value
**Independent effect of genotype on disease**			
**SNP1**	Mild	0.392	0.371
	Severe	0.245	0.407
**SNP4**	Mild	0.843	0.612

	Severe	1.556	0.229
**SNP5**	Mild	1.052	0.028*
	Severe	0.868	0.015*
**Effect of genotype-genotype interaction on disease**			
**SNP1:SNP4**	Mild	0.852	0.386
	Severe	0.009	0.991
**SNP1:SNP5**	Mild	-0.118	0.829
	Severe	0.110	0.797
**SNP4:SNP5**	Mild	-0.849	0.502
	Severe	-1.757	0.127
**Effect of protein-protein interaction on disease**			
**CCL2:CCL5**	Mild	-0.005	-
	Severe	-0.005	-
**Effect of genotype-protein interaction on disease**			
**SNP1:CCL2**	Mild	0.010	0.015*
	Severe	0.005	0.096
**SNP5:CCL2**	Mild	1.749178e-06	0.999
	Severe	1.652773e-03	0.683
**SNP4:CCL5**	Mild	0.020	0.703
	Severe	-0.001	0.973

Supplementary Table S6. Frequency distribution of SNPs in world populations based on 1000 Genome Project. Similar frequency distribution of rs1024611 and rs2280788 among Asian and the study populations probably reflects the scenario of JE disease burden for the countries. However, rs1799864 among the study population was observed to be comparatively than other populations

**SNP**	**rs1024611A**	**rs2857656G**	**rs2280788G**	**rs2107538C**	**rs1799864G**	**rs1799987A**
**African**	0.24	0.44	0.01	0.43	0.2	0.59
**American**	0.46	0.47	0.01	0.25	0.19	0.43
**Asian**	0.61	0.61	0.11	0.33	0.22	0.56
**European**	0.33	0.32	0.01	0.17	0.10	0.46
**Assam**	0.66	0.76	0.07	0.65	0.70	0.44

Supplementary Figure S7. Gel electrophoresis images of genotyping for all the studied SNPs. Abbreviations: M, molecular size marker; bp, base pairs, NC, negative control.

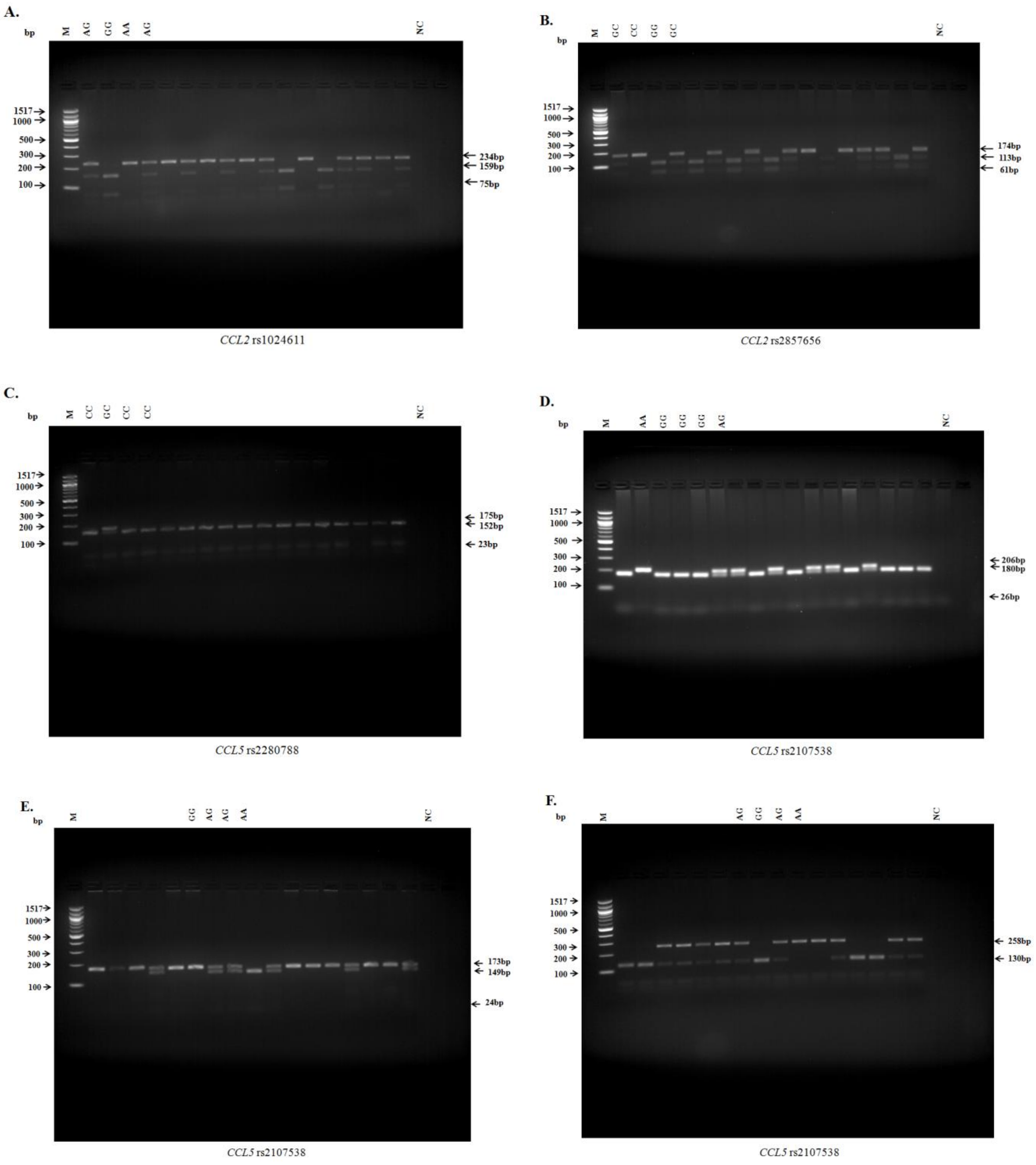

Supplement: Supplementary file 1 — supplementary data [file 41598_2017_14091_MOESM1_ESM.pdf]
